# Facial Surface Electromyography: A Novel Approach to Facial Nerve Functional Evaluation after Vestibular Schwannoma Surgery

**DOI:** 10.3390/jcm13020590

**Published:** 2024-01-19

**Authors:** Leonardo Franz, Gino Marioni, Antonio Daloiso, Elia Biancoli, Giulia Tealdo, Diego Cazzador, Piero Nicolai, Cosimo de Filippis, Elisabetta Zanoletti

**Affiliations:** 1 Phoniatrics and Audiology Unit, Department of Neuroscience (DNS), University of Padova, 31100 Treviso, Italy; gino.marioni@unipd.it (G.M.); cosimo.defilippis@unipd.it (C.d.F.); 2 Otolaryngology Unit, Department of Neuroscience (DNS), University of Padova, 35128 Padova, Italy; antonio.daloiso@aopd.veneto.it (A.D.); elia.biancoli@aopd.veneto.it (E.B.); giulia.tealdo@aopd.veneto.it (G.T.); diego.cazzador@aopd.veneto.it (D.C.); piero.nicolai@unipd.it (P.N.); elisabetta.zanoletti@unipd.it (E.Z.)

**Keywords:** surface electromyography, facial nerve palsy, vestibular schwannoma, synkinesis

## Abstract

Background: Vestibular schwannoma (VS) surgery may cause facial nerve damage. However, a comprehensive evaluation of post-operative facial outcomes may be difficult to achieve. Surface electromyography (sEMG) is a promising non-invasive evaluation tool. However, its use in the follow-up after VS surgery has not been reported yet. The main objective was to develop and validate a new sEMG application specifically for the post-VS surgery setting. Secondary goals were to provide a systematic description of facial muscle activity after VS surgery and assess the association between sEMG parameters and Sunnybrook scale scores. Methods: Thirty-three patients with facial palsy following VS surgery were included. The clinical outcomes (Sunnybrook symmetry, movement, and synkinesis scores) and sEMG parameters (signal amplitude normalized by the maximal voluntary contraction (NEMG) and sEMG synkinesis score (ESS, number of synkinesis per movement sequence)) were evaluated at the end of the follow-up. Results: In all tested muscles, NEMG variance was significantly higher on the affected side than the contralateral (variance ratio test, *p* < 0.00001 for each muscle). In total, 30 out of 33 patients (90.9%) showed an ESS ≥ 1 (median: 2.5, IQR: 1.5–3.0). On the affected side, NEMG values positively correlated with both dynamic and overall Sunnybrook scores (Spearman’s model, *p* < 0.05 for each muscle, except orbicularis oculi). ESS significantly correlated with the Sunnybrook synkinesis score (Spearman’s rho: 0.8268, *p* < 0.0001). Conclusions: We described and preliminarily validated a novel multiparametric sEMG approach based on both signal amplitude and synkinesis evaluation specifically for oto-neurosurgery. Large-scale studies are mandatory to further characterize the semiological and prognostic value of facial sEMG.

## 1. Introduction

Otoneurologic skull-base procedures, especially vestibular schwannoma (VS) surgery, are associated with a risk of facial nerve damage. Overall, this event is estimated to occur in about 15% of patients [1,2], potentially resulting in permanent functional and aesthetic sequelae in a non-negligible number of cases [3,4].

Several factors may affect the possibility of intraoperative facial nerve damage, including (i) the anatomical relationship between a tumor and nerve, (ii) the presence of a cleavage plane at the tumor interface, (iii) the need for extensive dissection maneuvers, and (iv) tumor size at the cerebello-pontine angle [3,5,6,7,8,9]. Recent meta-analyses [1,2] reported a post-operative loss of facial symmetry in about 6% of patients with Koos class [10] I–II schwannomas and in up to 50% of cases with Koos class III–IV.

A comprehensive and objective clinical evaluation of facial functional outcomes is usually hardly achievable during the post-operative follow-up of skull-base surgical procedures. Indeed, besides the gross evaluation of dynamic and static symmetry, which can be addressed by well-known clinical grading scales, there are subtler and, therefore, less immediately objectionable parameters inherent to the spontaneity of emotional movements or the presence of synkinesis [11,12]. As a result, there is currently no universally accepted gold standard method to evaluate facial nerve function in the post-operative setting. Moreover, the most common grading systems, including the House–Brackmann [13] and Sunnybrook [14] scales, are burdened by their subjective nature, thus potentially affecting the comparability of data. Therefore, the development of clinical tools to report the severity of facial nerve dysfunction in detail and assess it over time (especially in the post-operative follow-up) remains an open field, with a potential impact on diagnosis and rehabilitation strategies.

Electrophysiological techniques may allow an objective and reproducible evaluation of facial nerve function. However, the classical techniques, such as electroneurography and needle electromyography, are invasive and require a dedicated setting. As a result, they are difficult to use in a routine office setting. Although only a few reports are available [15,16], the assessment of facial function by surface electromyography (sEMG) represents a promising non-invasive solution to achieve objective follow-up data due to the absence of discomfort and the possibility of performing the examination in an outpatient clinic [17]. Nevertheless, data on the use of sEMG in the follow-up of iatrogenic lesions of the facial nerve secondary to skull-base surgery are still lacking.

The main aim of this study was to develop and preliminarily validate a new application of sEMG and a novel signal analysis protocol specifically for the clinical evaluation of facial nerve function in patients undergoing otoneurologic and skull-base surgery. Secondary goals were to (i) provide a systematic description of the sEMG activity of facial muscles in patients with facial nerve damage (and possible reinnervation) following VS surgery; and (ii) assess the association between quantitative electromyographic parameters and clinical features according to the Sunnybrook scale score.

## 2. Materials and Methods

### 2.1. Study Population

This study was conducted in accordance with the principles of the Helsinki Declaration. Data were examined in accordance with the Italian privacy and sensitive data laws and the Padova University Otolaryngology Section, Lateral Skull Base Unit internal rules. Before undergoing surgery, all patients included in this study signed a detailed informed consent form.

In this retrospective analysis, a consecutive series of patients with post-operative facial nerve palsy (Sunnybrook scale ≤ 99), following surgical resection of VS (via trans-labyrinthine, retro-sigmoid, or middle cranial fossa approaches), who underwent sEMG at their last follow-up, was considered.

Patients with a previous history of skull base and parotid irradiation or previous parotid surgery were excluded, as well as those with a diagnosis of type 2 neurofibromatosis. 

The first post-operative clinical evaluation of facial nerve function was performed one week after surgery and then two weeks later. 

Further follow-up controls were performed according to the degree of paralysis at least six months and one year after surgery. The function of the facial nerve was clinically quantified through the Sunnybrook scale [13,14]. A contrast-enhanced MRI obtained after at least six months was used to assess the completeness of the resection. 

At the last follow-up control, an sEMG (see Section 2.2) was also performed.

### 2.2. Surface Electromyography (sEMG)

A multi-channel Wave Plus Wireless EMG device with bipolar Mini Wave Infinity sensors (Cometa Systems, Bareggio, Italy) was employed. Each sensor was equipped with Ag/AgCl Kendall ARBO H124SG electrodes (Cardinal Health, Dublin, OH, USA).

To reduce cross-talk and maximize the signal-to-noise ratio, the inter-electrode distance was set at 8 mm, orienting the electrodes parallel to the fibers of the tested muscle [18,19]. To minimize electric impedance, the facial skin was prepared with an alcohol solution and shaved, if necessary. Each muscle group was tested simultaneously on both the affected and contralateral healthy sides.

**Figure 1:**
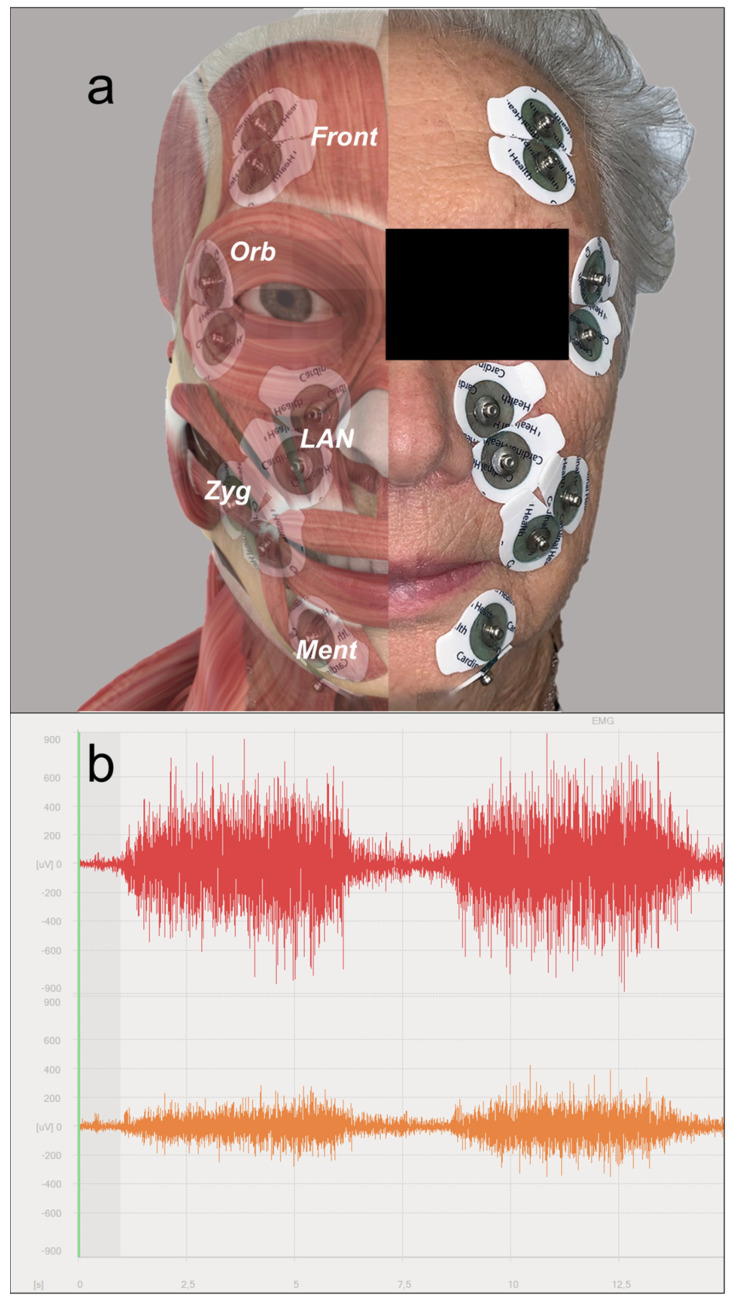
(**a**) Position of surface electrodes with reference to the tested muscles (Front: frontalis; Orb: orbicularis oculi; LAN: levator labii alaeque nasi; Zyg: zygomatic; Ment: mentalis); (**b**) example of raw sEMG signal (from levator labii alaeque nasi muscles): myogenic potential amplitudes appear to be lower on the affected side (in orange) compared to the healthy one (in red).

The tested muscles (see also Figure 1a), with their relative movement tasks (as derived from the Sunnybrook scale sequence [14]), were as follows:

1. Mentalis (lip pucker);
2. Levator labii alaeque nasi (snarl);
3. Zygomatic/risorius (open-mouth smile);
4. Orbicularis oculi (eye closure);
5. Frontalis (forehead wrinkle).

During sEMG evaluation, each movement was performed at the maximum voluntary contraction level, achievable without recruiting other neighboring muscle groups, and repeated three times. To evaluate synkinesis on the affected side by sEMG, a three-channel set-up was employed, simultaneously testing the upper, middle, and lower thirds of the face while the same movement sequence was performed.

### 2.3. Analysis of the EMG Signal

sEMG data were quantitatively analyzed using the EMG and Motion Tools Software 8.0 (Cometa Systems, Bareggio, Italy). 

To evaluate the symmetry of sEMG patterns in each muscle group, data from the affected and contralateral sides were analyzed simultaneously. To remove artifacts, the raw sEMG signal (see also Figure 1b) was filtered with a second-order high-pass filter, set at 10 Hz. 

Then, the time frame associated with the sEMG signal of voluntary contractions was identified. The sEMG signal underwent a smoothing process based on amplitude root mean square (RMS).

For normalization purposes, the maximal voluntary contraction (MVC) value was identified on the healthy side for each muscle in each patient, considering the RMS of the sEMG signal at the 500 ms time frame associated with the highest amplitude [18]. The integral of the sEMG signal (IEMG) over the voluntary contraction time, divided by the sampling frame length, was normalized by the MVC value of that specific muscle in that individual. The so-obtained value represented the normalized amplitude of the sEMG signal (NEMG) of each muscle, defined as a percentage of the MVC.

The presence of synkinesis was defined by identifying simultaneous sEMG activity compatible with muscle contraction in two or three channels during the movement sequence described in Section 2.2. The number of movements causing synkinesis per movement sequence (ranging from 0 to 5) was defined as the EMG synkinesis score (ESS).

### 2.4. Statistical Analysis

The distribution of NEMG values for each muscle was tested for normality by using the Shapiro–Wilk test. 

The Mann–Whitney and Kruskal–Wallis tests were used to compare the distribution of continuous variables, while Fisher’s exact test was used for categorical variables. 

The variation ratio test was applied to evaluate the difference in the distribution of NEMG variance between healthy and affected sides.

Asymmetry index (AI), considered the ratio between the difference and the sum of normalized sEMG values from the healthy (NEMG_h_) and affected (NEMG_a_) sides, was also calculated as follows:

$$AI=\frac{{NEMG}_{h}-{NEMG}_{a}}{{NEMG}_{h}+{NEMG}_{a}}$$

AI values could be positive in the case of the predominance of the healthy side or negative if the affected side showed hyperactivation.

Spearman’s model was used to test the correlations.

For graphical plotting of correlation analysis, fitted values and relative confidence intervals were based on the least-squares regression model.

A sample size analysis was performed to obtain data on the statistical power of Spearman’s correlation model. Assuming an alpha error of 0.05, a statistical power of 0.8, and a correlation coefficient of approximately 0.5 (indicating at least a “moderate” correlation degree), the necessary sample size was estimated to be at least 29 patients.

A cluster analysis was also conducted to identify sEMG patterns based on both NEMG and ESS to be tested for association with clinical features. The clustering method was based on K-median partitioning to classify cases based on their sEMG features (NEMG and ESS). The following modalities were applied: number of classes = 4 (using the Calinsky/Harabasz pseudo-F values to assess the stopping rule); measure of dissimilarity (Gower); initial group centers (k unique, random); and maximum number of iterations (set at 10,000) [20]. 

Statistical analyses were performed using Stata 16.1 (College Station, TX, USA).

## 3. Results

### 3.1. Clinical Features and Outcomes

Thirty-three consecutive patients were included. The preoperative facial nerve function was normal (Sunnybrook score: 100) in 29 cases and impaired (Sunnybrook score ≤ 99) in 4.

Twenty-seven patients underwent surgery via a trans-labyrinthine approach, while the approach to four patients was retro-sigmoid, one was trans-otic, and one patient was operated on via the middle cranial fossa route. The median tumor size in the cerebello-pontine angle was 2 cm (IQR: 1.6–2.2 cm). 

In seven cases, the intraoperative evidence of an anatomical facial nerve section led to a direct reconstruction by a great auricular (five cases) or sural (two cases) nerve cable graft. 

At the first post-operative evaluation (seven days after surgery), the median Sunnybrook score was 38 (IQR: 18–70). At the last evaluation, after a mean follow-up of 17.4 ± 39.8 months, the median Sunnybrook score was 53 (IQR: 32–92). At the last follow-up, two patients showed a Sunnybrook score of 100, while five had a score ≥ 95.

At the first post-operative evaluation, the difference in terms of the Sunnybrook score between patients undergoing nerve reconstruction with a graft and those not experiencing gross anatomical damage was not significant (Mann–Whitney U test: *p* = 0.1581). However, at the last follow-up, Sunnybrook scores were significantly lower in patients requiring a graft (Mann–Whitney U test: *p* = 0.0300).

### 3.2. sEMG Patterns on the Affected Hemiface and on the Healthy Side

**Figure 2:**
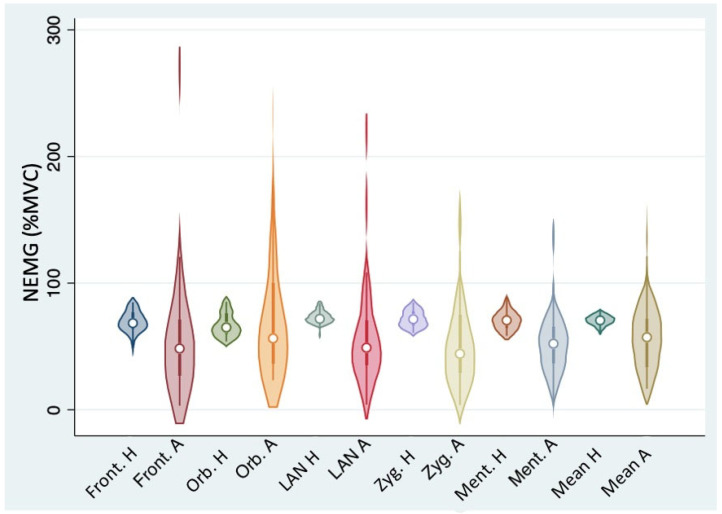
Mean and individual NEMG values for each muscle on both healthy and affected sides (Front: frontalis; Orb: orbicularis oculi; LAN: levator labii alaeque nasi; Zyg: zygomatic; Ment: mentalis; H: healthy side; A: affected side).

The distribution of NEMG values was normal on the healthy side for each tested muscle, except for the orbicularis oculi, which showed a non-normal distribution (Table 1). On the other hand, no muscle from the affected side showed any normal distribution of NEMG values (Table 1). Similarly, considering the average NEMG values calculated on the whole hemiface, their distribution was normal on the healthy side and non-normal on the affected side (Shapiro–Wilk test: *p* = 0.64738 and *p* = 0.01703, respectively). In all tested muscles, the variance of NEMG values was significantly higher on the affected side compared to the contralateral (variance ratio test, *p* < 0.00001 for every tested muscle, see Table 1 and Figure 2). 

Again, the asymmetry indexes (AIs) showed a substantially normal distribution on the healthy side, except for the orbicularis oculi, which demonstrated a trend towards a non-normal distribution (Shapiro–Wilk test: *p* = 0.05603).

At multi-channel sEMG evaluation, 30 out of 33 patients (90.9%) showed evidence of at least one synkinesis phenomenon per movement sequence (ESS ≥ 1). The median ESS was 2.5 (IQR: 1.5–3.0).

**Figure 3:**
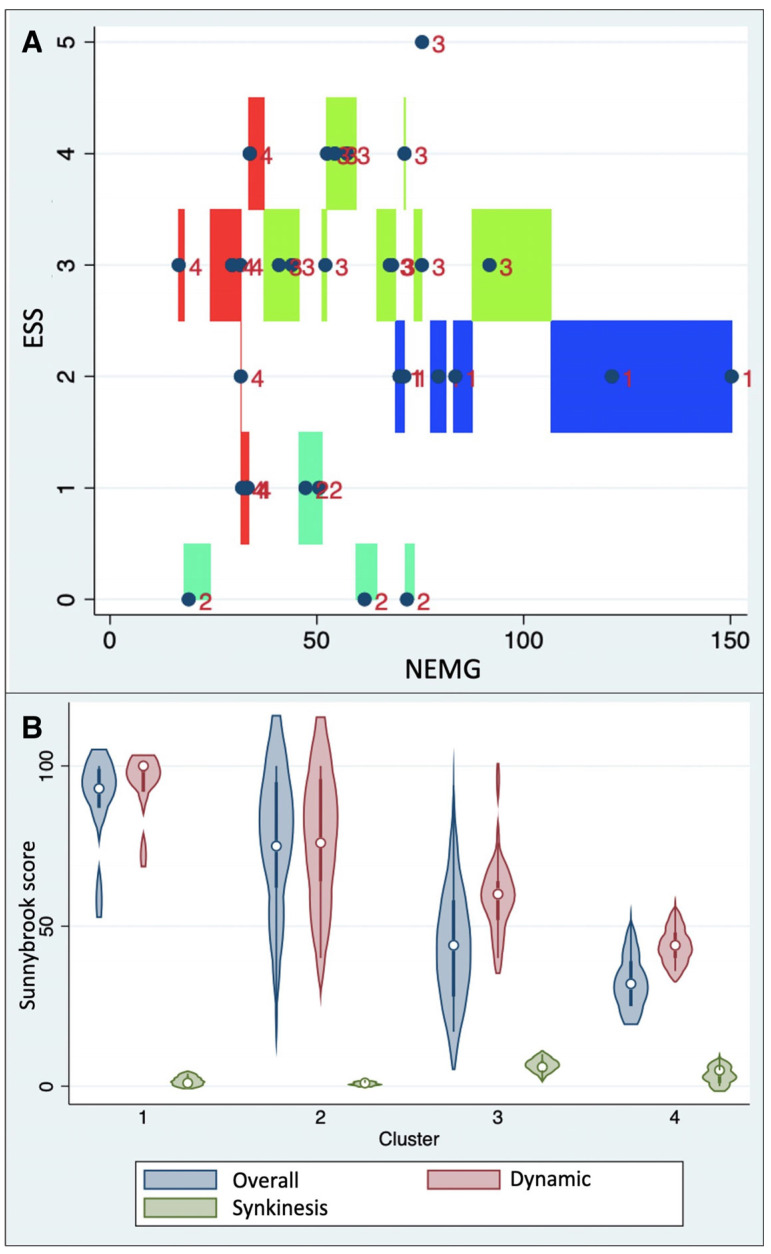
(**A**) Distribution of clusters with reference to the NEMG values and the ESS (cluster 1: blue; cluster 2: turquoise; cluster 3: green; cluster 4: red); (**B**) violin plot showing Sunnybrook scores (dynamic, synkinesis, and overall) stratified by cluster.

The cluster analysis based on NEMG and ESS led to the identification of four different clusters as follows (Figure 3A):

- Cluster 1 (median NEMG: 79.4% IQR 70.0–121.2%; median ESS: 2.0, IQR: 2.0–2.0);
- Cluster 2 (median NEMG: 50.5% IQR 47.3–61.5%; median ESS: 0.0, IQR: 0.0–1.0);
- Cluster 3 (median NEMG: 62.5% IQR 52.3–73.3%; median ESS: 3.0, IQR: 3.0–4.0);
- Cluster 4 (median NEMG: 37.8% IQR 30.5–33.1%; median ESS: 2.5, IQR: 1.0–3.0).

Calinski–Harabasz’s pseudo-F value, based on this four-class clustering model, was 18.15, indicating good inter-cluster discrimination.

### 3.3. Correlation between Sunnybrook Scores and Quantitative sEMG Parameters

**Figure 4:**
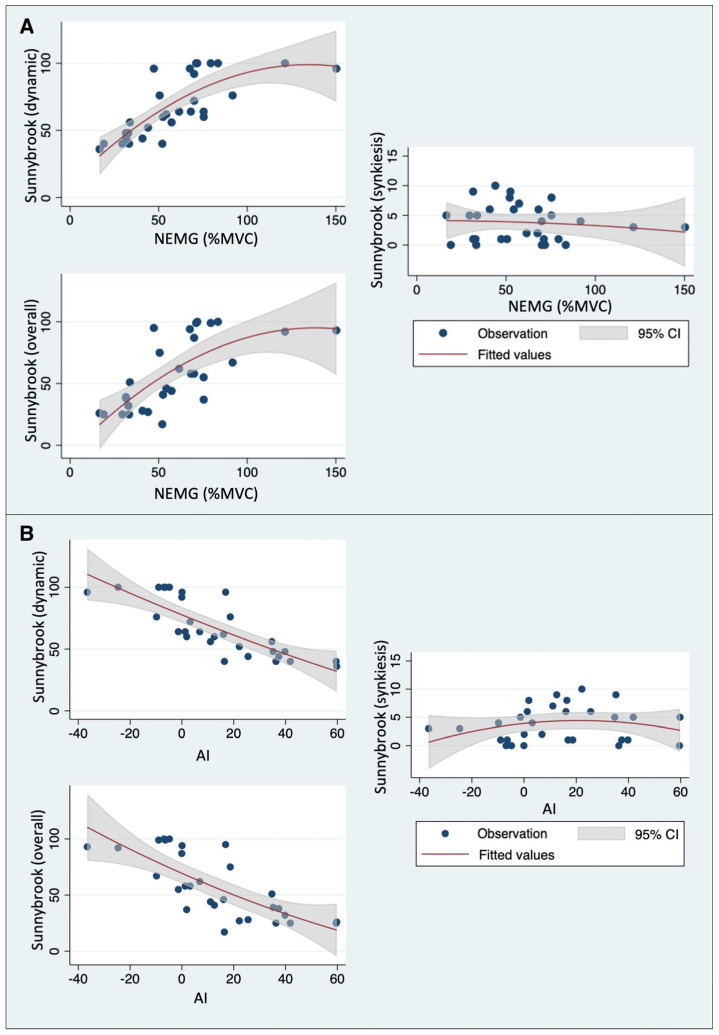
Association between Sunnybrook scores, (**A**) mean NEMG values, and (**B**) AI values. Fitted values and relative confidence intervals based on the least–squares regression model.

NEMG values on the affected side were positively correlated with both dynamic and overall Sunnybrook scores, whereas no correlation was found with synkinesis Sunnybrook scores (Table 2 and Figure 4A).

Similar results were found regarding the correlation between the AI values and Sunnybrook scores (Table 2). In this case, the AI values were inversely correlated with both dynamic and overall Sunnybrook scores. Again, no correlation was found with synkinesis Sunnybrook scores (Table 2 and Figure 4B).

Considering the ESS, a significant positive correlation was found with the Sunnybrook synkinesis score (Spearman’s rho: 0.8268, *p* < 0.0001), while no correlation emerged with either dynamic or overall Sunnybrook scores (Spearman’s rho: −0.2687, *p* = 0.1511, and Spearman’s rho: −0.3047, *p* = 0.1015, respectively).

Regarding the afore-mentioned clusters (see Figure 3A), significant differences among them were found in terms of dynamic, synkinesis, and overall Sunnybrook scores (Kruskal–Wallis test: *p* = 0.0005, *p* = 0.0012, and *p* = 0.0021, respectively). In particular, cluster 4 was associated with the lowest overall Sunnybrook scores (median: 32, IQR 25–39), while cluster 1 showed the highest ones (median: 93, IQR 87–99). A similar distribution was found for dynamic Sunnybrook scores, which were lower in cluster 4 (median: 44, IQR 40–48) and higher in cluster 1 (median: 100, IQR 92–100).

Instead, regarding synkinesis Sunnybrook scores, the highest values were found in cluster 3 (median: 6, IQR 5–8), while the lowest were those in cluster 2 (median: 1, IQR: 0–1). Figure 3B summarizes the distribution of Sunnybrook scores by cluster.

### 3.4. Association between Quantitative sEMG Parameters and Clinical Features

Patients who had undergone facial nerve reconstruction with a graft showed lower NEMG values compared to those who had not (Mann–Whitney U test: *p* = 0.0063 for the frontalis; *p* = 0.0190 for the mentalis; and *p* = 0.0135 for the whole hemiface, respectively). Instead, such a difference was not significant for the orbicularis oculi, levator labii alaeque nasi, and zygomatic (Mann–Whitney U test: *p* = 0.6088, *p* = 0.1164, and *p* = 0.0716, respectively).

Similar outcomes also emerged for the AIs, which were significantly higher in patients with facial nerve grafts for the frontalis and mentalis muscles, as well as for the average values on the whole hemiface (Mann–Whitney U test: *p* = 0.0190, *p* = 0.0263, and *p* = 0.0224, respectively), while no significant differences were found for the orbicularis oculi, levator labii alaeque nasi, or zygomatic (Mann–Whitney U test: *p* = 0.9729, *p* = 0.1036, and *p* = 0.0549, respectively). The ESS was not significantly associated with grafting (Mann–Whitney U test: *p* = 0.3681).

Moreover, the rate of patients who had undergone a graft was higher in cluster 4 compared to the other ones (Fisher’s exact test, *p* = 0.0500). 

The time from surgery showed no significant association with NEMG values (Spearman test, *p* = 0.6908), while it was weakly but significantly correlated with the ESS (Spearman’s rho: 0.411, *p* = 0.0194).

## 4. Discussion

This investigation first reported sEMG patterns of facial muscles in a homogeneous series of patients showing post-operative facial nerve damage after VS surgery. In this research, we applied sEMG in a clinical setting different from previous experiences [15,21,22]. Indeed, these studies [15,21,22] included only acute facial nerve palsy cases, whereas our patients had relatively long-standing dysfunction, which could potentially have led to reinnervation [6,23]. Our results seem to reflect such complexity, showing a wide distribution of NEMG values on the affected side. This finding allegedly expresses the heterogeneity of functional facial nerve results after different degrees of reinnervation, ranging from almost complete residual flaccid palsy to marked hyperkinesis. On the other hand, the NEMG values on the unaffected hemiface were homogeneously centered on 70% of the MVC, with a very narrow variability range. If confirmed by larger-scale studies, this observation could lead to the definition of normality parameters for the NEMG values of facial muscles.

Interestingly, in this study, a significant positive correlation between mean NEMG values and both dynamic and overall Sunnybrook scores was found for each individual muscle, except for the orbicularis oculi. This is in keeping with a previous observation by Ryu et al. [16], who studied a different subset of patients and used a different sEMG analysis approach. Such results may be explained by the intrinsic difficulty in clinically scoring orbicularis oculi motor function. In fact, especially in young patients, a good palpebral closure can be achieved by the gravity effect even in cases of complete orbicularis oculi paralysis, potentially leading to an overestimation of the dynamic Sunnybrook scores.

Regarding the electromyographic definition of synkinesis, in most cases (30 out of 33 patients), at least one event per movement sequence was found, showing that sEMG evaluation may be highly sensitive in detecting even subclinical synkinesis, possibly inapparent during standard clinical evaluation [24,25]. However, in our population, this fact did not lead to a significant mismatch between the clinical and electromyographic evaluation, allowing for a substantial correlation between the synkinesis Sunnybrook score and ESS. Moreover, ESS was found to be positively correlated with the time elapsed from surgery, indicating that patients with a more advanced reinnervation process showed more severe synkinesis.

To provide a comprehensive description of facial function using sEMG, we developed a multi-parameter approach according to the philosophy of multi-dimensional clinical assessment tools, such as the Sunnybrook, Sydney, and eFACE systems [14,24,25,26,27,28,29]. As a result, in our investigation, the amplitude of myoelectric activity was considered along with synkinesis, identifying, by means of a cluster analysis, four electrophysiological scenarios with different distributions of NEMG values and ESS. From our preliminary data, the cluster characterized by both low NEMG values and high ESS (cluster 4) was associated with the lowest overall Sunnybrook scores. Interestingly, all patients who had severe intraoperative facial nerve damage requiring graft reconstruction fell into that cluster. Instead, cluster 1 represented the clinically most successful group, being characterized by both a high NEMG and a relatively low ESS. Clusters 2 and 3, characterized by low NEMG and ESS and relatively high NEMG and high ESS, respectively, showed intermediate Sunnybrook scores (slightly worse in cluster 3). However, to clinically validate this clustering system, reference ranges for NEMG and ESS values need to be assessed by further studies on a wider scale. Our results could be a possible starting point for the clinical application of sEMG in the follow-up of patients with iatrogenic intracranial facial nerve damage, potentially allowing an objective definition of the clinical picture based on electrophysiological parameters. 

Other methods to obtain an objective assessment of facial function, based on the calculation of linear distances between anatomical landmarks or computer analysis of face pictures, have been proposed in the literature [30,31,32]. However, they are not widespread in clinical practice. Besides these attempts to quantify facial symmetry, electrophysiological techniques have long been employed to assess patients with facial nerve impairment. Such methods, including nerve excitability testing, electroneurography, blink reflex testing, needle EMG, and transcranial magnetic stimulation, have become part of the clinical repertoire to address different aspects of facial nerve dysfunctions, thus obtaining both diagnostic and prognostic data [24]. The interpretation of data provided by such diagnostic tools may rely on either qualitative or quantitative evaluation of the output signal in response to either direct electric stimulation (as for nerve excitability testing and electroneurography) or a volitional movement (as for EMG) [24]. Compared to other methods, EMG techniques allow an evaluation of the motor unit action potentials either directly from the muscle itself (in the case of needle EMG) or from the skin surface (in the case of sEMG) [17]. The potential advantages of the sEMG approach include its ease of use and the absence of invasiveness, which may make this technique a possible complement to clinical assessment in the follow-up of patients undergoing neuro-otological surgery. Moreover, the possibility of obtaining quantitative data might allow a less operator-dependent definition of facial nerve function, thus facilitating the comparison of functional outcomes and potentially offering a tool to monitor rehabilitation therapy results over time. In this sense, facial sEMG may find an ideal application setting in the otolaryngological field, potentially applicable to the follow-up of different conditions associated with facial nerve impairment, including inflammatory diseases (such as Bell’s palsy and Ramsay-Hunt syndrome), traumatic injuries, and post-surgical damage from a wide range of procedures, including middle ear, parotid, and skull-base surgery [24]. 

The main limitation of this study lies in its retrospective monocentric design and its relatively limited sample size, suggesting the need for caution in generalizing these results. However, in this study, the population size was adequate to obtain sufficient statistical power for most of the employed tests, particularly regarding correlation analysis, thus providing enough confidence in the statistical results. However, compared to other available studies [15,21,22,33], this research also has some strengths: (i) the substantial homogeneity of the patients included, who all had facial nerve impairment after surgical removal of vestibular schwannoma; (ii) the use of a quantitative analysis method based on normalization by MVC, allowing to easily interpret and compare the activation level of each studied muscle [18,34]; (iii) the development of a multiparametric analysis system based on both sEMG signal amplitude and the number of synkinesis per movement sequence; and (iv) the use of the electrophysiological correlates of the parameters considered by the Sunnybrook scale, thus allowing an unbiased correlation between clinical and sEMG data. Moreover, the use of this objective and quantitative methodology to assess the functional outcomes of facial nerve injuries may be generalized to other clinical scenarios, allowing users to (i) compare functional results in patients with facial nerve damage from different origins; (ii) obtain more accurate follow-up data for both clinical and research purposes; and (iii) develop novel quantitative metrics with a potential semiological value.

## 5. Conclusions

In this investigation, we described and preliminarily validated a novel multiparametric system based on both sEMG signal amplitude and synkinesis evaluation, specifically developed for oto-neurosurgery. 

Large-scale prospective studies are needed to further characterize the semiological and prognostic value of the quantitative aspects of the sEMG signal, with the potential aim of helping to select the type and timing of post-operative rehabilitation in patients with facial palsy after oto-neurosurgical procedures.

## Figures and Tables

**Table 1:** Mean and individual NEMG values for each muscle, on both healthy and affected sides.

Muscle	NEMG, Healthy Side (% of the MVC) Mean ± SD	NEMG, Affected Side (% of the MVC) Mean ± SD	Variance Ratio Test *p*-Value
Frontalis	68.9 ± 9.3	55.2 ± 49.6	<0.001
Orbicularis oculi	67.9 ± 10.1	73.5 ± 50.6	<0.001
Levator labii alaeque nasi	72.7 ± 5.9	59.0 ± 43.2	<0.001
Zygomatic	72.0 ± 7.0	58.5 ± 41.5	<0.001
Mentalis	70.6 ± 7.6	55.1 ± 29.1	<0.001
**Mean values**	**70.5 ± 4.2**	**59.7 ± 28.3**	**<0.001**

MVC: maximal voluntary contraction; SD: standard deviation.

**Table 2:** Correlation between sEMG parameters (NEMG and AI values) and Sunnybrook scores.

	Muscle	Dynamic Sunnybrook Score		Synkinesis Sunnybrook Score		Overall Sunnybrook Score	
		Spearman’s ρ	*p*-Value	Spearman’s ρ	*p*-Value	Spearman’s ρ	*p*-Value
**NEMG**	Frontalis	0.7106	**<0.0001**	−0.1712	0.3658	0.6410	**0.0001**
	Orbicularis oculi	0.4446	**0.0260**	0.0465	0.8254	0.3594	0.0776
	Levator labii alaeque nasi	0.6179	**0.0003**	0.0491	0.7966	0.5225	**0.0031**
	Zygomatic	0.5081	**0.0041**	−0.1788	0.3445	0.4335	**0.0167**
	Mentalis	0.5919	**0.0006**	−0.1830	0.3329	0.5263	**0.0028**
	**Mean values**	0.8220	**<0.0001**	−0.1232	0.5168	0.7231	**<0.0001**
**AI**	Frontalis	−0.7565	**<0.0001**	0.2091	0.2675	−0.7073	**0.0001**
	Orbicularis oculi	−0.3944	0.0511	−0.0724	0.7308	−0.3290	0.1083
	Levator labii alaeque nasi	−0.6331	**0.0002**	−0.0079	0.9672	−0.5424	**0.0020**
	Zygomatic	−0.5586	**0.0013**	0.2129	0.2587	−0.4845	**0.0067**
	Mentalis	−0.6297	**0.0002**	0.1747	0.3557	−0.5717	**0.0010**
	**Mean values**	−0.8640	**<0.0001**	0.1424	0.4527	−0.7839	**<0.0001**

## Data Availability

The data presented in this study are available on request from the corresponding author.
